# Smart Pediatric Oncology Tracker of Symptoms (SPOTS), a Web-Based Interface for the Pediatric PRO-CTCAE: Development and Usability Study

**DOI:** 10.2196/87821

**Published:** 2026-05-12

**Authors:** Stacey Crane, Deevakar Rogith, Melody Hellsten, Andrew D Miller, Jacqueline Castillo, Brandy Boeger, Susan Vidaurre, Stefanie Pieper, Cecile Nguyen, Duc Anh Thanh Huynh, Jessica Wooden, Amy Franklin, Karen Moody, Wenyaw Chan, Pamela S Hinds, Constance Johnson

**Affiliations:** 1 Cizik School of Nursing The University of Texas Health Science Center at Houston Houston, TX United States; 2 McWilliams School of Biomedical Informatics The University of Texas Health Science Center at Houston Houston, TX United States; 3 Department of Pediatrics Baylor College of Medicine Houston, TX United States; 4 Luddy School of Informatics, Computing, and Engineering Indiana University Indianapolis Indianapolis, IN United States; 5 Department of Pediatrics Patient Care The University of Texas MD Anderson Cancer Center Houston, TX United States; 6 School of Public Health The University of Texas Health Science Center at Houston Houston, TX United States; 7 Department of Nursing Science, Professional Practice and Quality Children's National Hospital, School of Medicine and Health Sciences George Washington University Washington DC, DC United States

**Keywords:** child, patient-reported outcome measures, system design, usability testing, co-design, symptom assessment, toxicities, adverse events, website, cancer, digital health

## Abstract

**Background:**

Children undergoing cancer treatment experience a range of treatment-related toxicities that significantly affect quality of life and adherence to therapy. Current methods for symptom reporting rely heavily on clinician interpretation of caregiver or child verbal reports, which can result in incomplete or inaccurate records. The Pediatric Patient-Reported Outcomes version of the Common Terminology Criteria for Adverse Events (Pediatric PRO-CTCAE; National Cancer Institute) provides a validated mechanism for direct symptom reporting by children and caregivers, yet its traditional administration and preselection of questions limit the breadth of symptom capture.

**Objective:**

This research aimed to co-design and conduct formative usability testing of the Smart Pediatric Oncology Tracker of Symptoms (SPOTS), a novel, web-based interface for the Pediatric PRO-CTCAE to allow children with cancer and their caregivers to comprehensively report symptoms.

**Methods:**

The research comprised 2 sequential phases: co-design and usability testing. Guided by child-computer interaction theory and participatory design methods, child-caregiver dyads collaborated with the research team to iteratively design and refine the SPOTS prototype. Nine participant dyads engaged in up to 3 co-design sessions that informed system features, layout, and content. During the usability phase, 12 additional dyads (6 with children aged 7-12 years and 6 with adolescents aged 13-17 years, each with a caregiver) completed structured usability tasks using the SPOTS prototype. Task completion, pathway efficiency, and user feedback were recorded through screen capture, field notes, and think-aloud protocols. Quantitative data were analyzed descriptively, and qualitative feedback was analyzed thematically.

**Results:**

SPOTS was described by users as “very clear” and “easy to navigate.” Participants valued the visual design, the use of a customizable character, and the opportunity for children to report symptoms independently. Key usability challenges included confusing terminology, navigation redundancy, and visual complexities. Quantitative task analyses indicated that while most structured tasks were completed successfully, many required excess steps or assistance. When not directed to use a specific screen, participants’ symptom reporting methods varied, with caregivers and adolescents preferring the Body Parts Screen and younger children favoring the Search Screen.

**Conclusions:**

The formative development of SPOTS demonstrates the feasibility and value of co-designing pediatric health technologies directly with children and caregivers. SPOTS has the potential to enhance the implementation of the Pediatric PRO-CTCAE by offering an engaging, child-friendly digital format that facilitates more direct symptom reporting. Future work will include a pilot study to further assess real-world usability, the quality of symptom capture (ie, completeness and accuracy), and integration with clinical workflows.

## Introduction

Cancer remains the second leading cause of death among children aged 1 to 19 years [1]. Treatment can extend for up to 3 years, substantially disrupting normal childhood development [2-4]. Treatment-related toxicities impose a significant burden on children with cancer and can compromise their ability to tolerate and complete therapy [5,6]. Symptomatic toxicities (ie, symptoms) are typically documented based on physicians’ interpretations of spontaneous verbal reports from children or their caregivers, which may not accurately reflect the children’s lived experiences [7]. Furthermore, the current documentation of symptomatic toxicities is difficult to retrieve, often incomplete, and limits our ability to examine individual and population-level symptom trends [2]. This is particularly problematic for children with cancer, as childhood cancer is a rare disease. Each toxicity that is not captured is a lost opportunity to understand the symptom experience, reduce suffering, and improve the health outcomes of children with cancer. A crucial step in improving data collection in pediatric oncology is to use child (self) and/or caregiver (proxy) reports to assess symptomatic toxicities, the gold standard for symptom assessment [6,7].

The Pediatric Patient-Reported Outcome Common Terminology Criteria for Adverse Events (Pediatric PRO-CTCAE; National Cancer Institute) is a tool developed by Drs Pamela S Hinds and Bryce B Reeve to improve the assessment of symptoms during clinical trials. This instrument captures subjective symptomatic toxicities via direct report from children or caregivers (based on the child’s age and cognitive ability) [8-10]. Both the self and proxy versions of the Pediatric-PRO CTCAE have established reliability, validity, and responsiveness, and can be used separately or concurrently with both children (ages 7-17 years) and/or adult caregivers [10-12]. The Pediatric PRO-CTCAE assesses 62 symptomatic toxicities using 1 to 3 survey questions per toxicity, for a total of 130 items. Because of its length, it is typically implemented in research studies using preselected toxicities of interest, which limits the range of symptoms that can be reported [11,13].

To broaden data collection from the Pediatric PRO-CTCAE beyond preselected toxicities of interest, it is necessary to develop an easy-to-use, child- and caregiver-friendly interface that can be used anywhere. Smart Pediatric Oncology Tracker of Symptoms (SPOTS) is a novel web-based interface for the Pediatric PRO-CTCAE developed by this study team [14]. The purpose of the SPOTS interface is to provide a method for systematically reporting all the symptoms a child experiences. In contrast to a traditional survey approach, this system does not require that children and/or caregivers respond to questions for all 62 symptomatic toxicities each time a report is made, nor does it limit users to solely reporting on preselected toxicities. Instead, SPOTS enables children and caregivers to report all the symptomatic toxicities relevant to the child’s experience.

The objective of this study was to conduct preliminary co-design and initial usability testing of SPOTS in collaboration with children with cancer and their caregivers. Guided by child-computer interaction theory, participatory design, and action research principles [15-17], the study engaged end users as active design partners throughout the development process. Current literature emphasizes the importance of co-design and participatory research in health care to improve acceptance and sustainability of new processes and to ultimately enhance patient outcomes [16-22]. Despite the challenges of co-designing with children, incorporating the unique insights of children and their caregivers is crucial when developing technology for their use [23-25]. When intended system users are included as design partners in a project, they significantly impact the design process and enhance the usability of the technology [18,26,27].

## Methods

### Overview of SPOTS

SPOTS is primarily a point-and-click web-based interface for the Pediatric PRO-CTCAE items. All symptomatic toxicities included in the Pediatric PRO-CTCAE can be reported in SPOTS. SPOTS was created for use on mobile devices but also functions on tablets and desktop computers.

**Figure 1:**
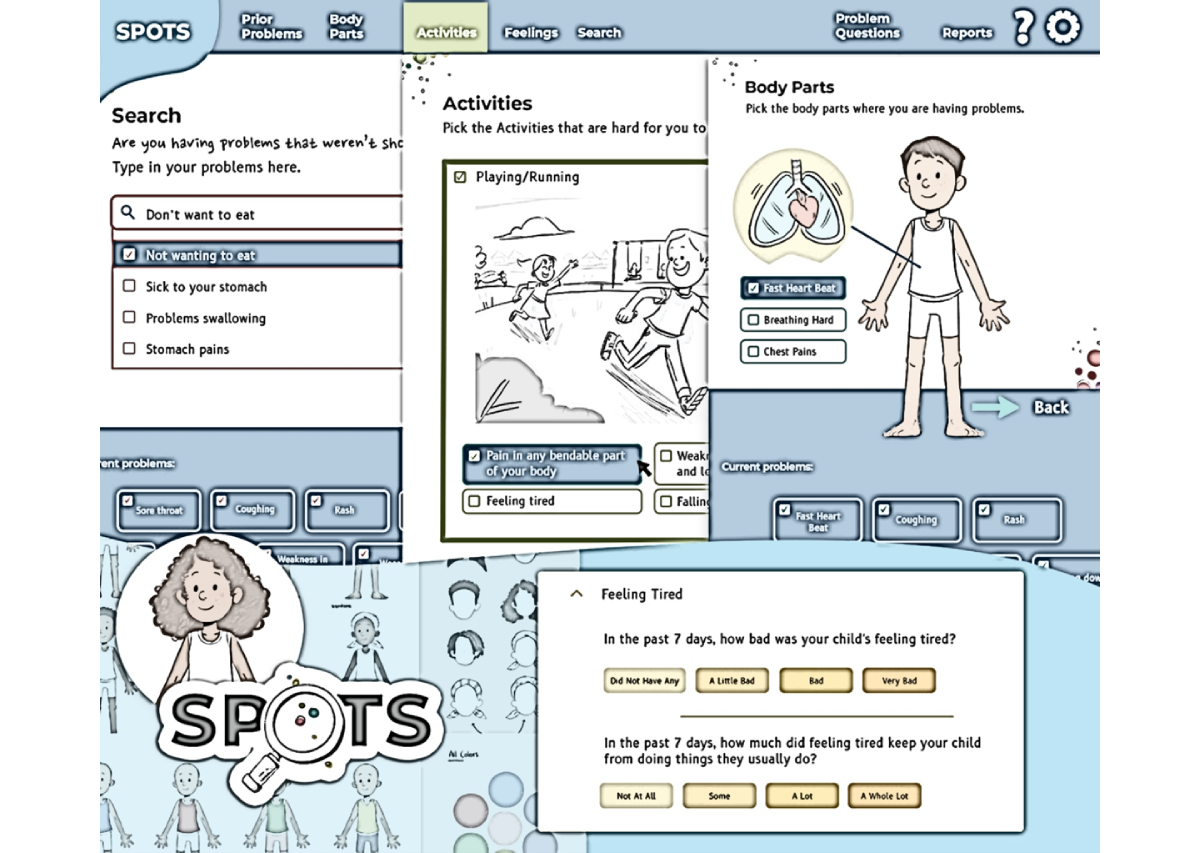
Screenshots of an early prototype of the Smart Pediatric Oncology Tracker of Symptoms (SPOTS) system.

SPOTS allows children and caregivers (eg, parents, guardians, or grandparents) to report symptoms in 5 different ways: based on previously reported problems, affected body parts, activity difficulties, feelings, and by using a search function (Figure 1). Users can apply any combination of these methods to identify symptomatic toxicities, aided by images, branching logic, and prompts. The search feature uses a lookup table to facilitate matching symptoms to appropriate Pediatric PRO-CTCAE items, while unmatched symptoms can be recorded as new symptoms. Children and caregivers can also personalize the SPOTS character’s appearance.

After identifying a symptom, the child or caregiver is presented with the applicable Pediatric PRO-CTCAE questions. Symptoms entered in SPOTS are used to generate symptom reports. While enrolled in a SPOTS study, automated weekly reminders are sent to participants via text or email to encourage regular reporting. If new or worsening moderate-to-severe symptoms are reported in SPOTS, a pop-up advises the user to seek medical evaluation [28].

### Development of SPOTS

We first developed a prototype for the SPOTS system, informed by our preliminary work [14]. This was followed by expert review of the content and wireframes to identify areas of refinement. Based on this evaluation, we designed and conducted additional co-design sessions to further inform system development, and subsequently integrated participant feedback into the SPOTS platform.

Next, we developed a usability testing protocol (including keystroke analysis) and conducted usability testing with end users. The results were analyzed, and relevant feedback was incorporated into the final design of SPOTS.

This paper reports findings from the co-design and usability testing phases specifically.

### Participants

Children with cancer and their caregivers were recruited as dyads through a private research participant recruitment firm and the University of Texas MD Anderson Cancer Center for the co-design phase, and through Texas Children’s Hospital for the usability phase. Clinic and inpatient census were reviewed by a study team member and prevetted participant lists by the firm, to identify potentially eligible participants. Recruitment was primarily conducted by providing information regarding the study and directing potential participants to contact either the study team or the firm for further details. Potential participants who contacted the firm were directed to the study team once the participant’s interest was verified. Children and caregivers were purposively selected for the approach based on the child’s treatment intensity (defined by Wolfe et al [29] and reported at enrollment via the Family Demographic Form) and the demographic characteristics needed to enhance the diversity of the accrued sample (ie, child gender and child age).

Inclusion criteria for child-caregiver dyads were (1) child age 7 to 17 years and caregiver age 18 years or older, (2) child diagnosis of any type of cancer more than 3 months prior to study enrollment, (3) child having received cancer treatment within the 12 months preceding study enrollment, (4) having access to a computer with a camera and internet access for video conferences, (5) willingness and ability to provide feedback in the design of SPOTS, and (6) English speaking. Children in foster care or who were wards of the state were excluded from participating.

After signing the informed consent and assent forms, the caregivers completed a brief Family Demographic Form, and the children completed a baseline Pediatric PRO-CTCAE Assessment. Questions on the Family Demographic Form included the child’s age, gender, race, ethnicity, and type of cancer; family configuration; household income; how often electronics (smartphone, tablet, and computer) are used by the caregiver and child; caregiver education level, gender, race, and ethnicity. This Pediatric PRO-CTCAE Assessment encompassed all questions related to the 15 core symptomatic toxicities on the Pediatric PRO-CTCAE [8,11,30]. The demographic information and symptom assessments were used to describe the sample and inform purposive sampling efforts.

### Co-Design Phase

#### Sample Size

Sample size in co-design research is determined as the study progresses, based on the heterogeneity of feedback provided. Typical recruitment of a co-design study ranges from 5 to 30 participants [17,23,31-36]. Convenience sampling was used in the co-design phase. Recruitment continued until feedback became redundant and the preliminary interface design was completed.

#### Study Design

Over the course of 12 months, participants attended up to 3 co-design sessions, with the number of sessions varying depending on the progress of interface design and the productivity of prior sessions. Sessions were scheduled based on the availability of participants and the study team, with caregivers and children participating together in the sessions. Sessions were conducted via videoconferencing and were video-recorded.

Co-design sessions were iterative and guided by child-computer interaction theory, which emphasized designing technologies that align with children’s developmental abilities; participatory design, which involves end users as active partners throughout the design process; and action research methods, which use cycles of reflection and refinement to improve the system and its implementation [15,16,20,22,23,31-33,37-39]. Sessions were led by the principal investigator (SC) or a trained study team member. To achieve a collaborative partnership with participants, techniques described by Druin [23] were used to set expectations for sessions and to strive for a neutral power structure (using first names, wearing informal clothing, avoiding hand-raising, and reimbursing all participants, including children, for their time). Sessions began with informal discussions and/or ice-breaking activities, then proceeded with telling, making, and/or acting activities [23,34,39]. Refer to Multimedia Appendix 1 for the co-design session interview guide, which included questions about the child’s symptom experience, feedback on website images, a symptom-to-picture matching activity, and questions regarding the website design.

### Usability Testing Phase

#### Sample Size

Our recruitment goal for the usability testing phase was 6 child-caregiver dyads in each of the following cohorts: dyads with children aged 7-12 years and dyads with adolescents aged 13-17 years. The sample size was determined based on recommended sample sizes from the literature, with the goal of minimizing the risk of overlooking usability problems while respecting the time and resources available [40-42]. Usability testing was planned with a total of 24 participants (12 caregivers, 6 children in the younger cohort, and 6 adolescents in the older cohort). Participants were purposively recruited to ensure representation from households with lower income levels.

#### Study Design

Next, usability testing was conducted on the prototypes developed during the co-design phase [43]. Sessions were held in-person in a private space within the clinic. During sessions, participants completed activities using a provided mobile device (ie, iPod Touch or iPhone). Usability testing used screen recording software, which allowed the time and clicks or keystrokes needed to complete each task to be recorded. A study team member led the usability sessions, recorded field notes of observed participant problems, and encouraged participants to think aloud so that their feedback would be captured in the interview transcript [44].

During usability sessions, participants were instructed on SPOTS and then directed to use it to complete a series of structured tasks, including login and logout, entry of specified symptoms of varying intensity, and retrieval of symptom output reports. Symptoms reported during usability testing were selected from a predetermined list and did not represent symptoms the child was currently experiencing. Depending on the task, symptom severity was either preassigned or selected by the participant. Severity was captured using the relevant Pediatric PRO-CTCAE items, which assess symptom frequency and perceived intensity. Throughout the session, the study team member asked the child or caregiver open-ended questions to explore the difficulties they had completing tasks and whether anything unexpected or unwanted happened [45]. Counterbalancing was used throughout usability testing. Specifically, the order of the structured tasks was randomized between participants to minimize the risk that the sequence in which the tasks were completed would influence performance [46]. Refer to Multimedia Appendix 2 for the usability testing interview guide, which outlines the questions asked about the child’s symptom experience and the tasks participants were asked to complete based on various website scenarios.

### All Phases

Sessions with participants lasted no more than 90 minutes in all study phases. With younger children, sessions were shorter (<60 minutes) and involved breaks. In recognition of their time and effort, each participant received a US $75 gift card at the end of each co-design and usability testing session (ie, both the caregiver and the child received their own gift card).

### Data Analysis

A professional transcriptionist from a Health Information Portability and Accountability Act (HIPAA)–compliant service transcribed recorded sessions. A trained study team member verified the transcript for accuracy, made corrections, and deidentified it by removing names, locations, and other potentially identifiable details prior to data analysis. Data were managed using REDCap (Research Electronic Data Capture; Vanderbilt University), MS Excel (Microsoft Corp), and MaxQDA (VERBI Software). Due to small sample sizes, quantitative data were analyzed with descriptive statistics.

Qualitative data were analyzed per Braun and Clark [47] method for latent, deductive thematic analysis, as follows. Significant statements (ie, statements related to usability of the SPOTS system) were identified and then classified as positive, neutral, or negative [48]. Significant negative statements were reviewed further to distinguish usability problems, using the usability problem categorization approach described by Zhang et al [48]. Usability problems were defined as “any aspect of the system that makes it unpleasant, inefficient, difficult, confusing, or impossible for the participant to achieve the [structured] tasks” [44], and encompassed suggestions for improvement.

Initial qualitative data analysis was conducted independently by 2 trained members of the study team. The 2 independent analyses were then reconciled by the first author and/or trained study team members. The first author reviewed any coding disputes and, when needed, established a consensus within the study team.

During data analysis, 2 trained study team members independently assigned a task completion score to each attempt at a structured task, as shown in Table 1 [49]. A keystroke analysis was also conducted. Specifically, screen recordings were independently analyzed by 2 trained members of the study team. The pathway taken to complete each task was carefully documented, including the number of steps taken to complete the task. The study team members reviewed their analyses to resolve any discrepancies in task completion scores and task pathways. Any unreconciled items were then evaluated by the first author for final determination. The minimum number of steps required to complete each structured task was determined by the first author after reviewing all keystroke data.

**Table 1:** Task completion scores to assess the usability of Smart Pediatric Oncology Tracker of Symptoms (SPOTS; adapted from Schoen et al [49]).

Score	Score description
3	Task completed correctly and easily
2	Task performed with hesitation, minimal assistance (spelling and confirmation questions) or a single error
1	Achieved task with confusion, with significant assistance, or with multiple inappropriate clicks
0	Task not completed correctly OR Taking more than 60 seconds to complete the task

### Ethical Considerations

This study was conducted in accordance with the principles of the Declaration of Helsinki and was approved by The University of Texas Health Science Center at Houston Institutional Review Board (July 8, 2020; HSC-SN-20-0042). All participant data were stored in a password-protected database accessible only to the study team, and all transcripts were deidentified prior to analysis. Informed consent was obtained electronically (via REDCap) from caregivers, parents, or legal guardians, and assent was obtained from all participants younger than 18 years of age. Consent and assent procedures included permission for the publication of deidentified study data. Participants were compensated for their time and effort with a US $75 gift card at the conclusion of each co-design and usability testing session.

## Results

### Overview

In the co-design phase, 9 child-caregiver dyads were recruited. No attempt was made to recruit participants based on age cohorts in the co-design phase, and as a result, 8 dyads with children aged 7-12 years and 1 dyad with a child aged 13-17 years were recruited. In the usability testing phase, 12 child-caregiver dyads were recruited: 6 dyads with children aged 7-12 years and 6 dyads with children aged 13-17 years. One child aged 17 years became unwell at the beginning of the usability testing and was unable to provide usable data. Across both phases, the final sample included 21 caregivers and 20 children. Caregivers were predominantly women (20/21, 95.2%) and White (15/21, 71.4%). Children had diverse cancer diagnoses and were also mostly White (15/20, 75%), although gender was more evenly balanced (12/20, 60% women). Refer to Table 2 for participant demographics.

**Table 2:** Demographic characteristics of study participants.

Demographic		Co-design phase			Usability testing phase	
		Caregiver (n=9)	Child (n=9)	Caregiver (n=12)		Child (n=11)
**Gender, n (%)**						
	Women	9 (100)	5 (55.6)	11 (91.7)		7 (63.6)
	Men	0 (0)	4 (44.4)	1 (8.3)		4 (36.4)
**Ethnicity, n (%)**						
	Hispanic or Latino	2 (22.2)	2 (22.2)	10 (83.3)		11 (100)
	Not Hispanic or Latino	6 (66.7)	5 (55.6)	2 (16.7)		0 (0)
	No response	1 (11.1)	2 (22.2)	0 (0)		0 (0)
**Race, n (%)**						
	White	6 (66.7)	6 (66.7)	9 (75)		9 (81.8)
	Black	0 (0)	1 (11.1)	0 (0)		0 (0)
	Two or more races	2 (22.2)	1 (11.1)	0 (0)		0 (0)
	Other	0 (0)	0 (0)	1 (8.3)		1 (9.1)
	Unsure	0 (0)	0 (0)	1 (8.3)		0 (0)
	No response	1 (11.1)	1 (11.1)	1 (8.3)		1 (9.1)
**Current age (years), n (%)**						
	7-12	—^a^	8 (88.9)	—		6 (54.5)
	13-17	—	1 (11.1)	—		5 (45.5)
**Smartphone, tablet, or computer use, n (%)**						
	Daily	7 (77.8)	6 (66.7)	12 (100)		10 (90.9)
	Often	1 (11.1)	3 (33.3)	0 (0)		1 (9.1)
	Rarely	1 (11.1)	0 (0)	0 (0)		0 (0)
**Primary cancer, n (%)**						
	Solid tumor	—	2 (22.2)	—		1 (9.1)
	Lymphomas	—	1 (11.1)	—		3 (27.3)
	Leukemia	—	5 (55.6)	—		7 (63.6)
	No response	—	1 (11.1)	—		0 (0)
**Total annual household income (US $), n (%)**						
<40,000		2 (22.2)	—	5 (41.7)		—
40,000-59,999		2 (22.2)	—	1 (8.3)		—
60,000-99,999		2 (22.2)	—	2 (16.7)		—
≥100,000		2 (22.2)	—	2 (16.7)		—
Prefer not to answer		1 (11.1)	—	2 (16.7)		—
**Highest level of education, n (%)**						
	Less than 12th grade	1 (11.1)	—	0 (0)		—
	High school graduate	1 (11.1)	—	3 (25.0)		—
	Some college or professional training	1 (11.1)	—	5 (41.7)		—
	College graduate	3 (33.3)	—	3 (25.0)		—

^a^Not applicable.

### Qualitative Findings From Co-Design and Usability Phases

#### Overview

Qualitative findings were organized thematically around the following: login and home page, navigation and terminology, images, symptom selection and identification, symptom rating, and overall impressions. Table 3 contains qualitative data analysis exemplars, including positive and negative statements made about the safety and privacy, readability, proximity, and visual hierarchy of the SPOTS website. Table 4 provides a comprehensive summary of all qualitative feedback obtained regarding the SPOTS website. The feedback in Table 4 is not differentiated by role, as caregivers and children agreed on the strengths and weaknesses of SPOTS.

**Table 3:** Qualitative data analysis exemplars from participants during the co-design phase^a^.

Category	Positive statements	Negative statements	Usability problem classification
Login and home page	“We’re very clear on what it is asking, on what the page is for.” (U2, Caregiver)	“...I’d rather have her not have a Gmail...” (U6, Caregiver)	Control
Navigation and terminology	“So, five ways to tell you about my problem.” (U14, Caregiver)	“Yeah, but if I was like seven or five, I would not know what that word means.” (C5, Child)	Language
Symptom selection and identification	“...you just click on the little kid and Pazam! … And then like all the things that you would’ve … fill in is already there. “ (U6, Caregiver)	“When selecting the head part, it was kind of hard because it was like scalp, nose, or eye, or left eye.” (U4, Child)	Match
Symptom rating	“Like … lighter means less painful and darker means more, or if it does not apply.” (C4, Child)	“Maybe ‘does not apply’ should be white, the first thing, and then ‘bad’, and then it gets darker.” (C7, Caregiver)	Visibility

^a^C: co-design phase; U: usability phase.

**Table 4:** Summary of qualitative feedback on the Smart Pediatric Oncology Tracker of Symptoms (SPOTS) website during the co-design phase.

Website feature	Feedback
Login and home page	The login process was overall understood well. Users wanted to decrease reliance on external login platforms (ie, Google).
Navigation and terminology	Individual pathways to report symptoms were understood well. Some users expressed confusion with multiple pathways that led to the same outcome. Some users expressed that the terminology was unclear on the main navigation menu which caused friction in deciding where to navigate.
Symptom selection and identification	Desired symptoms were easily identified and selected. The selection of smaller features caused frustration in some users (ie, clicking on eyes on a face). Symptom identification and selection were difficult for some because terminology did not align with their everyday language. Some users encountered repeated questions when recording symptoms, leading to uncertainty about task completion. Some users expressed interest in a voice search feature, so that children who are still learning to read or write can search and enter symptoms.
Symptom rating	Users generally understood how to rate the severity of their symptoms. A few users encountered issues with the visual or logical order of levels and loss of user input. Some users had difficulty editing symptom ratings, and suggested color-coded ratings would help.
Overall impressions	User feedback was highly positive. Most of the participants found the website to be easy to use and felt it provided value. Some users were interested in avatar personalization options. Users requested a comment box to add explanations, thoughts, or questions to the symptom report. Addition of a short tutorial was suggested to improve navigation.

#### Overall Impressions

Across all the participants, SPOTS was described as “very clear” and “easy to navigate.” Caregivers appreciated its potential to help children communicate their symptoms more independently, while children reported that the visual interface made health discussions feel “less scary” and “more like a game.”

Common themes for improvement included increasing color and personalization (eg, character customization by skin tone or hair), adding gamification elements (eg, stars or points for logging in and completing tasks), simplifying terminology, clarifying navigation, and enhancing visual feedback when symptoms are reported. In particular, participants expressed enthusiasm for the idea of a summary report showing the child’s symptom profile over time that can be retrieved within SPOTS.

#### Login and Home Page

Participants generally found the initial login process familiar and manageable, particularly when the Google login option was visible. However, some children had difficulty entering their email address and required caregiver assistance. While the interface was described as “plain but nice,” several participants noted it appeared too white and lacked visual engagement. Caregivers and children suggested adding more color or artwork and ensuring the SPOTS logo was included on the Google login screen.

#### Navigation and Terminology

Both children and caregivers suggested more color and visual cues, such as facial expressions or icons, to enhance engagement. To improve navigation, participants frequently requested clearer menu groupings, larger titles to help orient users, and prompts to guide users sequentially through sections before proceeding to ratings. Several users expressed difficulty understanding the order of tasks, such as when to move from selecting symptoms to rating them.

Most participants understood key menu items such as “Feelings,” “Body Parts,” “Settings,” “Help,” and “Reports.” However, several children and caregivers found certain terms ambiguous, including “Problems,” “Past Problems,” and “Activities.” The label “Problems” was sometimes interpreted as “website problems” rather than physical or emotional symptoms. The term “Past Problems” caused confusion as children thought it might refer to medical records rather than previously experienced symptoms. Many participants recommended renaming this tab. The “Feelings” tab was well understood; children could easily identify emotions or sensations they were currently experiencing.

The “Activities” tab was often misinterpreted as fun or leisure activities rather than a place to identify symptoms based on activities that are hard to complete. A more descriptive label, such as “Physical Activities” or “Things That Are Hard to Do,” was suggested.

Participants largely understood how to use the character body map to indicate physical symptoms. However, some younger children expected to click directly on the affected body part rather than select from a list. Several participants misinterpreted the “Skin” button, believing it might change the character’s skin color, highlighting the need for improved labeling.

#### Images

Participants provided detailed feedback on the images used in SPOTS to improve clarity and representativeness. Several images were found to be confusing or unclear, including those depicting the throat, hair, and self-harm. Some images conveyed unintended emotions or actions. For example, lines under the eyes made the character appear tired, and one image intended to show eating was interpreted as a dislike of food. Participants recommended enhancing realism and expressiveness by adding features such as pimples to depict skin conditions, hair to certain images, and clearer gestures (eg, a shrug with raised hands to show confusion). They also suggested removing background distractions and adjusting clothing or posture to better reflect the intended actions or contexts.

#### Symptom Selection and Identification

Children demonstrated partial understanding of how to select current and past symptoms that they were asked to report, sometimes requiring adult guidance to interpret questions such as “Which problems are you still having?” Rewording and enlarging this prompt were suggested to improve clarity.

While colors and checkmarks were generally understood as indicators of selection, the graphic displaying the number of questions answered was often overlooked or misunderstood. Participants recommended using brighter or contrasting colors, bolding questions, and color-coding symptom categories.

Despite occasional confusion, most participants were able to successfully identify how to report symptoms. However, some participants expressed concern about missing symptom options or incomplete menus. The matching activity in the co-design phase allowed children to identify which symptoms they associate with different body parts and activities. This feedback was then incorporated into SPOTS by iteratively adjusting the menus. The symptoms that participants struggled to find in SPOTS included abdominal pain, abdominal distention, back pain, constipation, cough, depression, diarrhea, fatigue, headache, insomnia, nausea, neuropathy, sore throat, and vomiting. Some participants recommended adding an “Other” or “Symptom not listed” option to all menus to help guide users who were struggling to identify a specific symptom. The primary reason participants struggled to find these symptoms was that the symptoms were not located where participants expected. However, some participants experienced temporary technical problems with the SPOTS interface that limited Search results. Notably, there was one symptom that a child-caregiver dyad wanted to report, which is not included in the Pediatric PRO-CTCAE: anger.

#### Symptom Rating

Once explained, children generally understood the rating scale, particularly when caregivers related it to widely used clinical scales, such as the Wong-Baker FACES Pain Rating Scale [50]. However, several usability issues emerged, including confusion between “Not at All” and “Does not Apply.” Participants expressed a preference for more visual aids when rating symptoms, such as gradient colors, emojis, or numerical scales. Participants also recommended reordering symptom rating options from least severe to most severe. Finally, participants requested audio or visual feedback (eg, a “ding” sound or color change) when a symptom rating was saved.

### Quantitative Findings From the Usability Testing Phase

Two pieces of quantitative data were associated with each structured task attempted by participants. First, the assigned task completion score, and second, the count of the number of steps it took participants to complete the structured task (from the keystroke analysis). A task completion score of 0 or 1 indicated that the task was not successfully completed, whereas a score of 2 or 3 indicated successful completion (refer to Table 1 for details).

Task completion scores from the usability phase revealed that participants had a range of success with the structured tasks. Table 5 summarizes the tasks with the lowest task completion scores (mean score <2). The most difficult tasks for participants included reporting “peeing self on accident” (mean 0.0, SD 0.0, n=2), “pimples” (mean 1.0, SD 1.4, n=4), and “itchy skin” (mean 1.3, SD 1.0, n=4), but these were reported by a small number of participants. Other tasks with low mean completion scores and a high number of participant attempts included reporting “back pain using the Activities Screen” (mean 1.4, SD 1.1, n=23), and “feeling like throwing up today using the Body Parts Screen” (mean 1.7, SD 1.4, n=22).

**Table 5:** Most difficult tasks based on low completion scores (mean <2) or excess steps taken to complete (median >2 beyond minimum steps required) during usability testing^a^.

Task		Task completion scores			Number of excess steps taken to complete the task, above the minimum required		
Toxicity to report	Screen to use	Mean (SD)	Median (IQR)	n	Mean (SD)	Median (IQR)	n
*Urinary incontinence*	*Any*	*0.0 (0.0)*	*0 (0-0)*	*2*	—^b^	—	*0*
*Pimples*	*Any*	*1.0 (1.4)*	*0.5 (0-1.5)*	*4*	*5.5 (3.5)*	*5.5 (4.3-6.8)*	*2*
*Itchy skin*	*Any*	*1.3 (1.0)*	*1.5 (0.8-2)*	*4*	*3.0 (1.0)*	*3 (2.5-3.5)*	*3*
Nausea	Body Parts	1.7 (1.4)	2 (0-3)	22	2.8 (4.3)	1 (0-2.5)	16
*Back pain*	*Activities*	*1.4 (1.1)*	*1 (0.5-2)*	23	*6.1 (5.2)*	*6 (2-8)*	*17*
*Blurry vision*	*Any*	*1.8 (1.5)*	*2 (0.8-3)*	*4*	3.3 (4.2)	*2 (1-5)*	*3*
Sneezing	Any	2.0 (1.4)	2 (1.5-2.5)	2	5.0 (7.1)	5 (2.5-7.5)	2
Sweating	Any	2.0 (0.0)	2 (2-2)	2	3.5 (0.7)	3.5 (3.3-3.8)	2
Urine discoloration	Any	2.0 (1.4)	2 (1.5-2.5)	2	3.0 (4.2)	3 (1.5-4.5)	2
Hiccups	Any	2.3 (0.5)	2 (2-2.3)	4	3.0 (2.9)	2.5 (1.5-4)	4
Headache	Body Parts	2.3 (1.1)	3 (1-3)	21	4.1 (3.8)	3.5 (1-5.3)	20
Muscle pain	Any	2.5 (0.7)	2.5 (2.3-2.8)	2	3.5 (2.1)	3.5 (2.8-4.3)	2
Problems swallowing	Any	2.5 (0.7)	2.5 (2.3-2.8)	2	3.5 (2.1)	1.8-3.3	2

^a^Italicized font denotes tasks with low completion scores (mean <2) and excess steps taken to complete (median >2 beyond minimum steps required).

^b^Not applicable.

Task efficiency was measured through the pathway analysis by the number of excess steps required to complete a task. Because each structured task required a minimum number of steps (between 2 and 4 steps) to complete, data are reported based on the number of additional steps participants took for each task beyond the minimum required (eg, a participant who took 5 steps to complete a task that could have been completed in 3 steps, took 2 excess steps). Table 5 summarizes the tasks with the highest excess steps. Participants, on average (median), required more than 2 excess steps to complete tasks than the minimum required. The most inefficient task completed by a larger number of participants included “report back pain using the Activities Screen” (median 6.0, IQR 2-8, n=17). Participants reporting back pain on the Activities Screen struggled with both excess steps and low completion scores, indicating significant usability problems. In contrast, participants who reported headaches on the Body Parts Screen had overall higher task completion scores, although they did require excess steps (median 3.5, IQR 1-5.3, n=20) to report the headache. Table 5 is not differentiated by role, as caregivers’ and children’s task completion scores and excess steps to complete tasks were not noted to differ substantively.

When asked to “report back pain using the Activities Screen,” the majority of participants (15/23, 65.2%) were unable to complete the task on that screen and instead used the Body Parts Screen (n=13) or the Search Screen (n=2). For the remaining structured tasks where participants were instructed to use a specific screen, participants very often (269/336, 80.1%) completed the task on the indicated screen. Table 6 lists the screens participants used to report each symptom when they were directed to use a specific screen, differentiated by participants who were and were not successful in completing the task. As there were some differences noted in caregivers’ and children’s abilities to successfully report these symptoms, Table 6 presents caregivers’ and children’s data separately.

**Table 6:** Screen used to report symptoms when prompted to use a specific screen during usability testing. In columns presenting pairs of n (%) values, the first pair corresponds to children; the second pair, caregivers. Italicized values represent those participants who used a different screen than the one they were prompted to use.

Task		Participants with successful task completion score (2 or 3; 118, 35.1% children and 151, 44.9% caregivers)				Participants with unsuccessful task completion score (0 or 1; 34, 12.8% children and 24, 7.1% caregivers)				
Toxicity to report	Screen to use	Body parts, n (%)	Search, n (%)	Activities, n (%)	Feelings, n (%)	Body parts, n (%)	Search, n (%)	Activities, n (%)	Feelings, n (%)	Challenges experienced
Abdominal pain	Search (n=23)	—^a^	11 (47.8); 11 (47.8)	—	—	—	0 (0.0) ; 1 (4.3)	—	—	Kept exiting without saving answers to symptom questions. Difficulty spelling “stomach.”
Anxiety	Feelings (n=23)	—	—	—	10 (43.5) ; 11 (47.8)	—	—	—	1 (4.3); 1 (4.3)	Reported “depression” instead of “anxiety.”
Back pain	Activities (n=23)	*2 (8.7)*; *5 (21.7)*	*1 (4.3)*; *1 (4.3)*	1 (4.3); 0 (0.0)	—	*4 (17.4)*; 2 (8.7)	—	3 (13.0); 4 (17.4)	—	Wanted to specify pain location, instead of reporting as nonspecific “pain.”
Constipation	Activities (n=23)	—	** **—	9 (39.1); 10 (43.5)	** **—	** **—	*1 (4.3)*; *1 (4.3)*	1 (4.3); 1 (4.3)	—	Symptom appeared far down on page, made it difficult for participants to see questions.
Cough	Body Parts (n=23)	8 (34.8); 12 (52.2)	—	—	—	3 (13.0); 0 (0.0)	—	—	—	Difficulty determining which body part was associated with coughing.
Decreased appetite	Activities (n=21)	—	—	9 (42.9); 8 (38.1)	—	*1 (4.8)*; *0 (0.0)*	—	1 (4.8); 2 (9.5)	—	Reported another stomach-related symptom instead.
Diarrhea	Body Parts (n=23)	8 (34.8); 11 (47.8)	—	—	—	3 (13.0); 1 (4.3)	—	—	—	Did not know how to navigate to the back side of the character’s body.
Fatigue	Feelings (n=21)	—	—	—	8 (38.1); 8 (38.1)	—	—	—	2 (9.5); 3 (14.3)	Difficulty finding on Feelings screen due to interface error.
Headache	Body Parts (n=21)	4 (19.0); 10 (47.6)	—	—	—	6 (28.6); 1 (4.8)	—	—	—	Difficulty selecting the head instead of other facial features on the character.
Insomnia	Activities (n=23)	—	—	10 (43.5); 12 (52.2)	—	—	—	1 (4.3); 0 (0.0)	—	Kept exiting without saving answers to symptom questions.
Nausea	Body Parts (n=22)	5 (22.7); 8 (36.4)	—	—	—	5 (22.7); 4 (18.2)	—	—	—	Difficulty differentiating between “throwing up” and “feeling like throwing up.”
Neuropathy	Body Parts (n=23)	7 (30.4); 10 (43.5)	** **—	** **—	—	4 (17.4); 2 (8.7)	—	—	—	Difficulty understanding “tingling.” Children couldn’t tell left from right on character. Selected leg instead of foot.
Depression	Search (n=22)	—	10 (45.5); 7 (31.8)	—	*0 (0.0)*; *2 (9.1)*	—	0 (0.0); 2 (9.1)	—	*1 (4.5)*; *0 (0.0)*	Preferred reporting on the Feelings screen instead of the Search Screen.
Sore throat	Search (n=22)	—	7 (31.8); 12 (54.5)	—	—	—	3 (13.6); 0 (0.0)	—	—	Difficulty spelling “sore throat.”
Vomiting	Search (n=23)	*0 (0.0)*; *1 (4.3)*	8 (34.8); 11 (47.8)	—	—	—	3 (13.0); 0 (0.0)	—	—	Symptom not found using the search bar. Difficulty spelling “throwing up.”
Totals	(n=336)	34 (10.1); 57 (17.0)	37 (11.0); 42 (12.5)	29 (8.6); 30 (8.9)	18 (5.4); 21 (6.3)	26 (7.7); 10 (3.0)	7 (2.1); 4 (1.2)	6 (1.8); 7 (2.1)	4 (1.2); 4 (1.2)	**—**

^a^Not applicable.

When participants were not directed to use a specific screen, their methods for reporting symptoms varied. Overall, participants across all 3 groups (caregivers, children aged 7-12 years, and adolescents aged 13-17 years) showed a clear preference for the Body Parts Screen and the Search Screen. Caregivers most often used the Body Parts Screen (29/65, 44.6%) for younger children (ages 7-12 years), the Search Screen (13/26, 50%) and for adolescents (ages 13-17 years), the Body Parts Screen (14/26, 53.8%). Table 7 provides a summary of the screens participants used to report symptoms when they were not instructed to use a specific screen, differentiated by caregivers, older children, and adolescents.

**Table 7:** Screen used to report symptoms when given the option of using any screen during usability testing.

Screen used	Symptom reports, n (%)		
	Caregivers (n=12)	Children aged 7-12 years (n=6)	Adolescents aged 13-17 years (n=5)
Activities	5 (7.7)	2 (7.7)	5 (19.2)
Feelings	4 (6.2)	—^a^	1 (3.8)^b^
Body parts	29 (44.6)	11 (42.3)	14 (53.8)
Search	27 (41.5)	13 (50)	6 (23.1)
Total	65 (100)	26 (100)	26 (100)

^a^Children aged 7-12 years were not asked to report a symptom available on the Feelings screen.

^b^Adolescents aged 13-17 years were only asked to report 3 symptoms available on the Feelings screen.

## Discussion

### Principal Findings

Results from SPOTS’ formative development indicated that SPOTS was generally viewed as comprehensive, intuitive, and beneficial. Children and caregivers shared that symptom reporting felt more engaging and less intimidating through SPOTS. Participants provided valuable feedback to refine SPOTS, suggesting enhancements such as clearer terminology, improved navigation cues, gamification elements, and the need for summary reports of symptom trends. Usability testing confirmed that both children and caregivers could successfully complete key tasks using SPOTS, although some terminology and visual elements required simplification.

Well-designed interfaces significantly enhance the use of a system and the data captured, as the interface is how users visualize and interact with the system [51]. Indeed, poorly designed health information technology that fails to consider users’ needs often has unintended negative effects on efficiency, user satisfaction, and health care quality [51-54]. The work-centered co-design method used to develop SPOTS will facilitate its implementation in practice and enhance its usefulness long-term [55-57]. We found that child-computer interaction theory, participatory design, and action research methods each influenced SPOTS’ design in ways that helped reduce cognitive load and improve usability for children and caregivers. Child-computer interaction theory emphasizes aligning digital environments with children’s developmental abilities, attention spans, and prior experiences, and guided our design decisions toward familiar and intuitive system features [23,31-33]. Similarly, participatory design principles focus on engaging end users in iterative co-design and ensure that SPOTS fulfills the expectations of children and caregivers, thereby minimizing extraneous cognitive effort [16,22,38,39]. Action research methods further supported SPOTS’ usability through cycles of observation, reflection, and redesign, allowing usability issues to be identified and addressed in real time [15,20,37]. These theoretical and methodological foundations reflect well-established usability principles, which emphasize the importance of recognition over recall, consistency, and alignment with users’ mental models and expectations [58-60]. For instance, incorporating interface elements that were already familiar to users, such as a “hamburger” menu to represent navigation or a gear icon for settings, reduced cognitive load by not requiring users to learn new symbols or actions. By grounding its design in established theories and usability heuristics, we confirmed that SPOTS supports efficient and intuitive interaction, as well as decreased cognitive load during use.

The usability analysis revealed that both child and caregiver participants tended to associate site-specific symptoms with particular body parts and preferred the ability to report the same symptom for multiple body parts. For example, although “pain” could be reported in relation to various activities on the Activities Screen, most participants chose to report back pain using the Body Parts Screen. This pattern underscores the importance of modifying SPOTS to enable symptoms to be reported from multiple screens, rather than restricting each symptom to a single entry per login session. SPOTS’ original design, which is based on the structure of the Pediatric PRO-CTCAE, allows each symptom to be reported only once during a login session (eg, pain recorded once without direct linkage to a specific body part or screen, though the source menu is captured in the data). Participants’ preference for associating symptoms with Body Parts Screen may be influenced by the visual layout of the on-screen character and the Body Parts Screen, which likely reinforced the intuitive connection between physical symptoms and anatomical location [61-63].

Differences in developmental stage appeared to influence how children interacted with the system, even within a small sample. Despite challenges with spelling accuracy during symptom searches, approximately half of the younger children preferred to use the Search Screen to enter symptoms whereas more than half of adolescents favored the Body Parts option when given a choice. This unexpected finding suggests that differing cognitive strategies or levels of familiarity with using natural language search queries may influence how children of different ages prefer to navigate symptom reporting tools [64,65]. In future pilot testing, we plan to examine children’s search terms in greater detail to better understand their symptom conceptualization and language use. Additionally, children expressed confusion when asked to report symptoms by left or right body side, as they interacted with an on-screen character and were uncertain which side corresponded to their own. To reduce cognitive load and potential reporting errors, future iterations will remove left or right body side selection from the Body Parts page, instead collecting this information with the subsequent symptom-specific questions.

Although the symptomatic toxicities contained in the Pediatric PRO-CTCAE capture the majority of symptoms that participants wished to report, a notable gap emerged. One child-caregiver dyad expressed a desire to report anger, a symptom not represented in the existing toxicity items. While Pediatric PRO-CTCAE items include “sad or unhappy feelings” and “thinking about hurting yourself,” anger is a distinct emotional state and is closely associated with feelings of fear, grief, and loss of control—emotions commonly experienced by children undergoing cancer treatment and their caregivers [66-68]. In the absence of an appropriate category, children and caregivers wanting to report anger may default to recording it under “sad or unhappy feelings,” potentially leading to misclassification and an underrepresentation of this important emotional experience. However, the SPOTS platform provides an opportunity to capture missing elements from the Pediatric PRO-CTCAE by allowing users to search for symptoms and, if not found, enter what they are feeling as a new symptom. On the backend, these unmatched search entries are saved distinctly, along with the search string entered by the participant, and will continue to provide valuable insights into symptomatic toxicities that may be missing from the Pediatric PRO-CTCAE.

These study results highlight that electronic health systems that support children’s health should be designed iteratively and collaboratively, incorporating feedback from both children and caregivers throughout all development stages [24,32,69,70]. Engaging children as design partners ensures that the system is developmentally appropriate, intuitive, and visually engaging [23-25,71,72]. Despite our team’s best efforts during the development of the SPOTS website, insights from children and caregivers during the formative development of SPOTS highlighted how to further simplify website language, add visual cues to improve user flow, provide interactive user feedback, and offer greater flexibility during symptom entry.

The next step in SPOTS’ development is to conduct a preliminary longitudinal pilot study using the SPOTS prototype developed through this formative research. The goal of this pilot study will be to identify challenges with SPOTS’ use outside of the clinical setting. Future development of SPOTS will focus on refining the interface based on user feedback from the pilot study and expanding its functionality to support broader use. Planned enhancements include adding gamification features to encourage regular engagement, improving user visualization of symptom entry through longitudinal reports, streamlining ways to report symptoms, and creating a culturally and linguistically adapted Spanish version of SPOTS. Following further development, we will conduct larger-scale usability and feasibility testing to evaluate SPOTS’ impact on symptom communication and clinical care, as well as explore its integration within electronic health records.

Limitations of this formative work include the reliance on convenience sampling in the co-design phase and limited participant diversity, both of which may restrict generalizability. While attempts were made in the co-design phase to recruit participants from across the United States, participants were primarily recruited from a narrow geographic region, which may not fully capture the experiences of children and families from diverse cultural or socioeconomic backgrounds. The small sample sizes in this study also limited our ability to fully compare responses across age groups; however, future pilot testing will include a larger cohort and support more in-depth age-related analyses. Additionally, the prototypes developed represent early-stage versions of SPOTS, meaning that findings reflect preliminary feedback rather than performance of a robust system in real-world clinical use.

### Conclusions

This formative development research demonstrated that SPOTS provides a comprehensive and intuitive platform for the Pediatric PRO-CTCAE, enabling child and caregiver reports of symptomatic toxicities. The co-design methods used to develop SPOTS increase the system’s usability, sustainability, and acceptance in real-world health care settings. Future development will focus on assessing the quality of symptom capture (ie, completeness and accuracy), refining the interface based on real-world usage patterns, enhancing navigation features, incorporating gamification elements, translating the system into Spanish, and integrating it into clinical workflows.

## Data Availability

The datasets generated or analyzed during this study are not publicly available because they contain sensitive, potentially identifiable information from a small pediatric population, but are available from the corresponding author on reasonable request.

## Appendix

Multimedia Appendix 1 Co-design phase interview guide.

Multimedia Appendix 2 Usability testing phase interview guide.

## Abbreviations

HIPAA: Health Information Portability and Accountability Act
Pediatric PRO-CTCAE: Pediatric Patient-Reported Outcomes version of the Common Terminology Criteria for Adverse Events
REDCap: Research Electronic Data Capture
SPOTS: Smart Pediatric Oncology Tracker of Symptoms
