# Supplementary material for: Smart Pediatric Oncology Tracker of Symptoms (SPOTS), a Web-Based Interface for the Pediatric PRO-CTCAE: Development and Usability Study
Source: JMIR Form Res. 2026 May 12;10:e87821. doi: 10.2196/87821 (PMC13213329; doi:10.2196/87821)
Supplement: Multimedia Appendix 1 [file formative_v10i1e87821_app1.docx]

Co-Design Phase Interview Guide

1. Welcome
2. Complete Consent, Assent, Demographic and Symptom Assessment Forms
3. Icebreaker
4. Child’s Symptom Experience

- Ask the child only:
  - *What does the word ‘symptom’ mean?*
  - *Do you use other words instead of 'symptom'?*
  - *What are the symptoms that you/your child experience?*
  - *How do you currently track/share these symptoms?*
  - *What does/does not work about how you currently track/share symptoms?*
  - *Do you have any artwork or journals you would be willing to share that you kept while you/your child were in treatment?*

1. Image Questions

- Show the following in three distinct groups: pictures of body parts, activities, and feelings.
- Ask for each picture:
  - *What is this a picture of?*
  - When appropriate, ask: *What do / don’t you like about that picture?*
  - If child does not correctly identify the picture, say: *It is supposed to be a picture of XXX. How would you draw a picture of XXX?*
- After showing all of the activity pictures, ask: *These are pictures of common activities that kids do. What activities are missing that you do on a regular basis?*

1. Matching Activity

- There are 62 symptoms. The order for the symptoms to be presented was pre-selected prior to the session to ensure that all symptoms were assessed multiple times with a sample of participants. As many symptoms as possible were included in each session.
- Show the following in three distinct groups: picture of character, labeled pictures of activities, and unlabeled pictures of feelings.
- Ask for each symptom:
  - *Where would you put this symptom on these pictures? You can put it in as many places as you would like.*

1. Website Design Questions

- Introduce the website.
- Show each webpage. Ask for each webpage:
  - *What do you think you would do on this webpage?*
  - *What do/don’t you like about how this webpage looks?*
  - *Are the instructions clear? If not, how else could we word them?*
  - *Is it clear what is selected/not selected on this page?*
  - *What would you do if you wanted to undo/unselect what you had done?*
  - *What parts of this webpage would you change?*
  - *I would like you to XXX. Please show me how you would do that on this webpage.*
- *How useful do you think this website will be for reporting your/your child’s symptoms?*
- Describe the overall flow of the website, i.e., to first identify their symptoms and then to rate them.
  - *There are many different ways to identify symptoms in SPOTS and you can use any combination of them. Is that clear? How can we make that clearer?*
  - *How can we improve the overall flow of the website so that it is easy to understand what you are to do?*
  - *What method do you think you would primarily use to identify your symptoms?*
