# Supplementary material for: Smart Pediatric Oncology Tracker of Symptoms (SPOTS), a Web-Based Interface for the Pediatric PRO-CTCAE: Development and Usability Study
Source: JMIR Form Res. 2026 May 12;10:e87821. doi: 10.2196/87821 (PMC13213329; doi:10.2196/87821)
Supplement: Multimedia Appendix 2 [file formative_v10i1e87821_app2.docx]

SPOTS Usability Testing Phase Interview Guide

1. Welcome
2. Complete Consent, Assent, Demographic and Symptom Assessment Forms
3. Icebreaker
4. Child’s Symptom Experience

- Ask the child only:
  - *What does the word ‘symptom’ mean?*
  - *Do you use other words instead of 'symptom'?*
  - *What are the symptoms that you/your child experience?*
  - *How do you currently track/share these symptoms?*
  - *What does/does not work about how you currently track/share symptoms?*
  - *Do you have any artwork or journals you would be willing to share that you kept while you/your child were in treatment?*

1. Website Scenario-Based Questions

- Ask child and parent to try to complete the following tasks using the website.
  - Login.
  - Report symptoms of a particular intensity using a specific method per Table A below. Randomize the order using one of the random lists of numbers.
  - Review symptoms they recorded.
  - Edit symptoms they recorded:
    - Change to severe headache
    - Change to mild cough
    - Delete sore throat
  - Report additional symptoms using any method they wish. Randomly select the symptoms asked about from Table B below.
  - Logout

Table A: Symptoms of a Specified Intensity to Report Using a Specific Method

| # | Task | Screen to Use for Task |
| --- | --- | --- |
| 1 | Report mild headache | Body Part |
| 2 | Report bad cough | Body Part |
| 3 | Report throwing up twice yesterday | Search |
| 4 | Report feeling a little tired | Feelings |
| 5 | Report not eating much over the past few days | Activities |
| 6 | Report a lot of tingling in your right foot | Body Part |
| 7 | Report a really bad sore throat | Search |
| 8 | Report not being able to sleep last night | Activities |
| 9 | Report feeling like going to throw up today | Body Part |
| 10 | Report back pain | Activities |
| 11 | Report a lot of runny poop | Body Parts |
| 12 | Report feeling sad over the last week | Search |
| 13 | Report really bad stomach pains | Search |
| 14 | Report not being able to poop for five days | Activities |
| 15 | Report feeling a little worried | Feelings |

Table B: Symptoms of Any Intensity to Report Using Any Method

- Arms and legs feel weak
- Bigger belly than usual
- Bruise easily (get black and blue marks on your skin)
- Burning feeling in your chest (heartburn)
- Change in the color of your pee
- Changes in your voice
- Dizziness
- Dry eyes
- Dry mouth
- Dry skin
- Fall Down
- Fart more than usual
- Feel hot all of a sudden (hot flashes)
- Feel like you could not wait to pee
- Food or drink taste different than usual
- Hair fall out
- Hiccups
- Hoarse (scratchy) voice
- Itchy red bumps on your skin
- Itchy skin
- Muscles hurt
- Nose bleeds
- Not being able to sit still
- Open sores or red spots on your skin
- Pain in any bendable part of your body (knees, etc.)
- Pain or burning when you pee
- Pee more than usual
- Pee yourself on accident
- Pimples (bumps on the face or chest)
- Poop yourself on accident
- Problems breathing (shortness of breath)
- Problems remembering things
- Problems with paying attention (focusing on TV, reading, or school work)
- Problems with swallowing
- Puffiness (swelling) in your arms, hands, legs, or feet
- Racing heartbeat
- Ringing or buzzing in your ears
- Flashes of light that were not there when your eyes were open or closed
- See blurry (have blurry vision)
- Shaking chills
- Sneezing
- Sunburn more easily
- Sweat more than usual
- Think about hurting yourself
- Watery eyes (tearing)
- Wheezing (a whistling noise in your chest when you breathe
